# Cartilage Extracellular Matrix Homeostasis in Knee Osteoarthritis: Pathomechanisms and Therapeutic Targeting

**DOI:** 10.1111/jcmm.71384

**Published:** 2026-10-11

**Authors:** Chao Du, Jiwei Zhong, Jialin Yang, Yanbo Huang, Qi Liu, Dongdong Cao, Tianci Guo, Jida Wang, Aifeng Liu

**Affiliations:** ^1^ First Teaching Hospital of Tianjin University of Traditional Chinese Medicine Tianjin China; ^2^ National Clinical Research Center for Chinese Medicine Acupuncture and Moxibustion Tianjin China

**Keywords:** biomarkers, chondrocyte extracellular matrix, homeostatic imbalance, knee osteoarthritis, therapeutic strategies, tissue engineering

## Abstract

Knee osteoarthritis (KOA) is a degenerative joint disease primarily driven by the disruption of homeostasis in the chondrocyte extracellular matrix (CECM). This review systematically examines the central role of CECM homeostasis in KOA pathogenesis and summarizes recent advances in this field. We synthesized contemporary basic and clinical research to analyse the mechanisms underlying CECM homeostatic imbalance. These include biomechanical stress, inflammatory responses, programmed cell death (e.g., apoptosis, pyroptosis, ferroptosis), impaired autophagy, and cellular senescence. We also evaluated intrinsic risk factors and the application of related biomarkers for diagnosis and prognosis. Our analysis identifies multifaceted pathways driving CECM imbalance and highlights promising multi‐modal therapeutic strategies. These include novel pharmacological agents (e.g., high‐purity chondroitin sulphate, natural active monomers), physical and regenerative therapies (e.g., stem cells, platelet‐rich plasma), precision interventions (e.g., gene editing, RNA interference), and tissue engineering approaches (e.g., smart biomaterials, biomimetic scaffolds). The evidence demonstrates that these strategies can effectively modulate CECM metabolism, promote cartilage repair, and slow disease progression. Targeting CECM homeostasis is a core strategy for developing disease‐modifying therapies for KOA. Future efforts should focus on interdisciplinary integration, individualized precision medicine, and multi‐site synergistic interventions. This review provides a comprehensive foundation for innovating strategies to delay or reverse KOA progression by restoring CECM balance.

## 1 Introduction

Knee osteoarthritis (KOA) is a chronic and debilitating disease characterized by progressive degeneration of articular cartilage as its core pathological feature, affecting the quality of life for millions of people worldwide [1]. Traditionally viewed as a ‘wear and tear’ disorder, emerging research has increasingly revealed that its pathogenesis is an active process involving a complex interplay of mechanical, biological and cellular‐molecular factors [2]. Within this process, the homeostatic imbalance between the synthesis and degradation metabolism of the chondrocyte extracellular matrix (CECM) is considered the pivotal driver of cartilage degeneration, ultimately leading to the initiation and progression of KOA [3].

The CECM functions not as a passive scaffold but as a dynamic biological microenvironment, with its homeostasis being critical for maintaining the structural integrity and functional properties of cartilage tissue [4, 5]. In KOA, diverse pathological insults disrupt CECM metabolic equilibrium, resulting in hyperactivation of matrix‐degrading enzymes, substantial loss of crucial matrix components and initiation of self‐perpetuating pathological cascades [6, 7, 8, 9]. Although substantial research has concentrated on inflammatory pathways and chondrocyte apoptosis in KOA, comprehensive reviews systematically examining the molecular mechanisms and multimodal therapeutic strategies through the lens of CECM homeostasis regulation remain scarce.

Although several recent reviews have addressed cartilage extracellular matrix in osteoarthritis from various perspectives, most focus on a single dimension and fail to provide a systematic integration through the unifying framework of chondrocyte extracellular matrix homeostasis [10, 11, 12, 13, 14, 15, 16]. Existing reviews have not comprehensively covered the synergistic regulatory network among multiple programmed cell death modalities, impaired autophagy, and cellular senescence at the pathomechanistic level, nor have they integrated quantitative imaging biomarkers with traditional fluid biomarkers at the biomarker level. A systematic synthesis of multi‐tiered therapeutic strategies ranging from natural active monomers to smart biomaterials and from cell therapy to gene editing remains lacking, and the role of sex‐specific differences in chondrocyte extracellular matrix homeostatic imbalance has been rarely addressed. This review aims to fill these gaps by taking chondrocyte extracellular matrix homeostasis as the central thread, systematically analysing the complex molecular network underlying its imbalance in knee osteoarthritis, evaluating intrinsic risk factors that compromise this equilibrium, providing a parallel assessment of fluid and imaging biomarkers for dynamic monitoring, and highlighting emerging multi‐tiered therapeutic strategies including novel pharmacological agents, physical and regenerative therapies, precision interventions, and tissue engineering approaches. The review concludes by discussing challenges and future directions in interdisciplinary integration and clinical translation, ultimately providing a framework for developing precision medicine strategies for knee osteoarthritis through chondrocyte extracellular matrix homeostasis modulation.

## 2 Methods

### 2.1 Literature Search Strategy

We systematically searched PubMed and Web of Science databases for articles pertaining to chondrocyte extracellular matrix homeostasis in knee osteoarthritis and related therapeutic strategies, with the search period covering up to July 2026. The search terms were organized into three groups and combined using Boolean operators. The first group comprised disease‐related terms, including ‘Knee Osteoarthritis’, ‘Knee OA’, ‘Gonarthrosis’, ‘Knee Degenerative Joint Disease’, and the MeSH term ‘Osteoarthritis, Knee’. The second group comprised chondrocyte extracellular matrix‐related terms, including ‘Cartilage Extracellular Matrix’, ‘Cartilage ECM’, ‘Cartilage Matrix’, ‘Decellularized Cartilage Matrix’, ‘Decellularised Cartilage Matrix’ and ‘Decellularized Cartilage Extracellular Matrix’, along with MeSH term combinations such as ‘Extracellular Matrix’ combined with ‘Cartilage’ or ‘Cartilage, Articular’, and extended to include ‘Decellularized Extracellular Matrix’, ‘Tissue Scaffolds’, ‘Hydrogels’ and ‘Biomimetic Materials’ in combination with ‘Cartilage’. The third group comprised therapy‐related terms including ‘Treatment’, ‘Therapy’, ‘Repair’, ‘Regeneration’, ‘Regenerative Medicine’, ‘Tissue Engineering’, ‘Cartilage Repair’, ‘Cartilage Regeneration’, ‘Chondrogenesis’, ‘Osteochondral Repair’ and MeSH terms including ‘Tissue Engineering’, ‘Regenerative Medicine’ and ‘Osteoarthritis, Knee/therapy’.

### 2.2 Study Selection Criteria and Screening Process

The inclusion criteria were: (1) studies addressing knee osteoarthritis and chondrocyte extracellular matrix homeostasis; (2) document types including original research and (3) articles published in English or Chinese. The exclusion criteria were: (1) reviews, systematic reviews, conference abstracts, case reports, and articles for which the full text was unavailable; (2) studies not related to chondrocyte extracellular matrix homeostasis or not focused on knee osteoarthritis. Two independent reviewers screened titles and abstracts based on the inclusion criteria, and disagreements were resolved through discussion or consultation with a third reviewer. Through the above search strategy, combined with manual reference tracing and expert recommendations, a total of 49 articles meeting the criteria were ultimately included.

## 3 The Pathological Role of CECM in KOA

### 3.1 Composition, Function and Homeostasis of the CECM

The CECM is an essential component of cartilage tissue, playing a critical role in maintaining its normal structure and function. Its primary constituents include collagen, proteoglycans, and glycosaminoglycans, among others. Collagen confers strength and toughness to cartilage, with type II collagen serving as the predominant form that is crucial for maintaining cartilage architecture [17]. Proteoglycans, characterized by abundant glycosaminoglycan side chains, exhibit high hydrophilicity, enabling them to attract and retain water molecules, thereby allowing cartilage to withstand compressive loads and absorb impact forces. Beyond providing physical support for chondrocytes, the CECM actively participates in intercellular signalling by harbouring various growth factors and cytokines. These signalling molecules orchestrate chondrocyte proliferation, differentiation, and metabolic activities, thereby preserving cartilage homeostasis. Under physiological conditions, CECM synthesis and degradation remain in dynamic equilibrium, with transforming growth factor‐β (TGF‐β) playing a significant role in promoting CECM synthesis by chondrocytes, which is vital for cartilage development and repair.

### 3.2 Manifestations of CECM Homeostatic Imbalance in KOA

#### 3.2.1 Biomechanical Alterations

Chondrocytes sense mechanical forces through an array of mechanosensitive receptors and ion channels on their cell membrane [18]. Among these, Transient Receptor Potential Vanilloid 4 (TRPV4) is activated by physiologic levels of mechanical loading and promotes the expression of chondrogenic genes and the production of cartilaginous matrix proteins [19]. In contrast, Piezo1 is preferentially activated by supraphysiologic or excessive mechanical deformation and drives catabolic responses, cell death, and senescence. Notably, excessive mechanical stress upregulates Piezo1 expression in chondrocytes, which in turn activates the PI3K/AKT/mTORC1 pathway, leading to reduced synthesis of collagen II and aggrecan while promoting matrix degradation [20]. Mechanical overloading can also activate Piezo1 channels to increase Ca^2+^ influx and trigger ferroptosis via the PI3K/AKT signalling pathway.

In addition to ion channels, integrins serve as critical mechanoreceptors that mediate cell‐matrix adhesion and translate mechanical cues into intracellular biochemical signals [10]. Excessive mechanical stress activates integrins αVβ3 and αVβ5 on the chondrocyte surface, inducing inflammatory responses through phosphorylation of Focal Adhesion Kinase (FAK) and Mitogen‐Activated Protein Kinases (MAPKs), which upregulate the expression of interleukin‐1β (IL‐1β), tumour necrosis factor‐α (TNF‐α), matrix metalloproteinase‐3 (MMP‐3), and MMP‐13. The integrin‐FAK‐MAPK mechanotransduction cascade thus represents a key axis linking abnormal biomechanical loading to cartilage degradation.

These mechanotransduction pathways do not operate in isolation but intersect with the biological alterations described in the following section. The TRPV4‐GSK3β signalling axis, for instance, in human osteoarthritic chondrocytes, is impaired in osteoarthritic chondrocytes, rendering these cells unable to sense and respond to changes in ECM viscoelasticity [21]. This dysfunction creates a vicious cycle: aberrant mechanotransduction activates catabolic signalling and promotes ECM degradation, which in turn alters the mechanical microenvironment and further impairs chondrocyte mechanosensing, ultimately accelerating CECM degradation and KOA progression [18].

Because of these molecular and cellular disruptions, the composition and structure of the CECM undergo profound alterations. The primary manifestations include increased degradation of type II collagen and reduced proteoglycan content, which collectively diminish cartilage hydrophilicity and shock‐absorbing capacity, thereby compromising its mechanical integrity. These changes not only directly impair the load‐bearing and cushioning functions of cartilage but also trigger abnormal remodelling of the subchondral bone and promote osteophyte formation. This further alters the lower‐limb biomechanical environment, establishing a vicious cycle that drives KOA progression. The regulation of CECM homeostasis by mechanical loading extends beyond chondrocytes. The meniscus, as a critical load‐bearing structure in the knee, exhibits cellular responses that are equally pivotal. Ma et al. [22] demonstrated that short‐term mechanical unloading, simulated using a 3D human meniscus model under simulated microgravity conditions, significantly upregulates OA marker genes such as COL10A1, MMP13, and SPP1, while activating inflammatory, calcification, and Wnt signalling pathways. Their study further revealed notable sex‐specific differences: in female‐derived cells, the JUN protein functioned as a hub gene regulating proliferation and inflammatory pathways, whereas male‐derived cells predominantly regulated ECM components and remodelling enzymes. This discovery provides novel molecular insights into the higher prevalence of KOA in females and underscores the importance of considering sex‐specific strategies in both research and therapeutic development.

#### 3.2.2 Biological Alterations

In KOA, the biological alterations of the CECM are primarily characterized by an abnormally enhanced catabolism, activation of multiple programmed cell death (PCD) pathways, and disruption of key intracellular homeostatic mechanisms. These changes collectively exacerbate cartilage degeneration.

The degradation of the CECM is predominantly driven by increased expression of matrix metalloproteinases (MMPs) and a disintegrin and metalloproteinase with thrombospondin motifs (ADAMTSs). The aberrant changes in the CECM can further activate inflammatory signalling pathways within chondrocytes, leading to increased release of pro‐inflammatory cytokines, which in turn amplify the inflammatory response and aggravate cartilage damage. Beyond classical inflammatory cytokines, high mobility group box 1 (HMGB1), an important damage‐associated molecular pattern (DAMP), also plays a critical role in this process. Shu et al. [23] discovered that HMGB1, via activation of the PI3K/AKT/GSK‐3β/β‐catenin signalling pathway, significantly upregulates the expression of MMP‐2, MMP‐3, MMP‐9, MMP‐13, ADAMTS4, and ADAMTS5. This not only induces chondrocyte apoptosis but also promotes the expression of oestrogen sulfotransferase (EST‐1) and Runt‐related transcription factor 2 (Runx2), thereby accelerating cartilage matrix degradation and facilitating the pathological differentiation of chondrocytes.

Beyond apoptosis, other modalities of programmed cell death (PCD) are deeply involved in the disruption of CECM homeostasis. Pyroptosis, mediated by caspase‐1 activation, leads to cell rupture and the massive release of IL‐1β and IL‐18, profoundly exacerbating inflammation. Qian et al. [24] identified miR‐107 as a pivotal negative regulator. Downregulation of miR‐107 relieves its inhibition on caspase‐1, thereby triggering the pyroptosis pathway and accelerating type II collagen degradation. Conversely, overexpression of miR‐107 effectively suppresses this process. Ferroptosis represents another significant mechanism. In human OA cartilage samples and preclinical mouse models, Zhao et al. [25] revealed that the downregulation of Selenoprotein M (SELM), an endoplasmic reticulum (ER)‐resident protein, specifically activates the PERK/EIF2α/ATF4 signalling pathway. This activation leads to the upregulation of its downstream target gene CHAC1, which depletes intracellular glutathione (GSH), ultimately inducing ferroptosis and aggravating CECM degradation. Protein misfolding resulting from ER stress initiates this cascade; the loss of SELM impairs interactions with molecular chaperones, compromises the correct folding of ECM components, and the accumulation of misfolded proteins further amplifies the stress pathway.

Autophagy, a crucial mechanism for maintaining intracellular homeostasis, is functionally impaired in OA chondrocytes. This impairment leads to the accumulation of damaged organelles and protein aggregates, thereby accelerating cartilage degeneration. In human OA cartilage samples and preclinical mouse models, Chen et al. [26], through proteomic analysis, identified a significant upregulation of the small GTPase Rab1a in OA cartilage. Rab1a activates the mTORC1‐S6K signalling pathway, which in turn inhibits the function of key autophagy initiation complexes such as the ULK1 complex, thereby potently suppressing autophagic flux. This suppression results in increased chondrocyte apoptosis and accelerated CECM degradation. Conversely, targeted inhibition of Rab1a was shown to activate autophagy and delay OA progression.

Furthermore, oxidative stress serves as another critical factor disrupting CECM homeostasis. The excessive accumulation of reactive oxygen species (ROS) directly damages collagen and proteoglycans, activates matrix‐degrading enzymes while simultaneously inhibiting their synthesis, and also triggers chondrocyte apoptosis [27].

The senescent phenotype of chondrocytes is also closely associated with CECM degradation. In human OA cartilage samples and preclinical mouse models, Zhao et al. [28] discovered that fibroblast activation protein (FAP) is highly expressed in OA cartilage and promotes the release of senescence‐associated secretory phenotype (SASP) factors by activating the NF‐κB pathway, thereby accelerating CECM degradation. In contrast, Collins et al. [29] demonstrated that the activity of the nuclear deacetylase sirtuin 6 (SIRT6) is significantly reduced in senescent chondrocytes. This SIRT6 deficiency suppresses the expression of insulin‐like growth factor‐1 (IGF‐1) and impairs the downstream Akt signalling pathway, leading to decreased synthesis of key CECM components such as type II collagen alpha‐1 chain (COL2A1) and consequently exacerbating OA progression (Figure 1).

**FIGURE 1:**
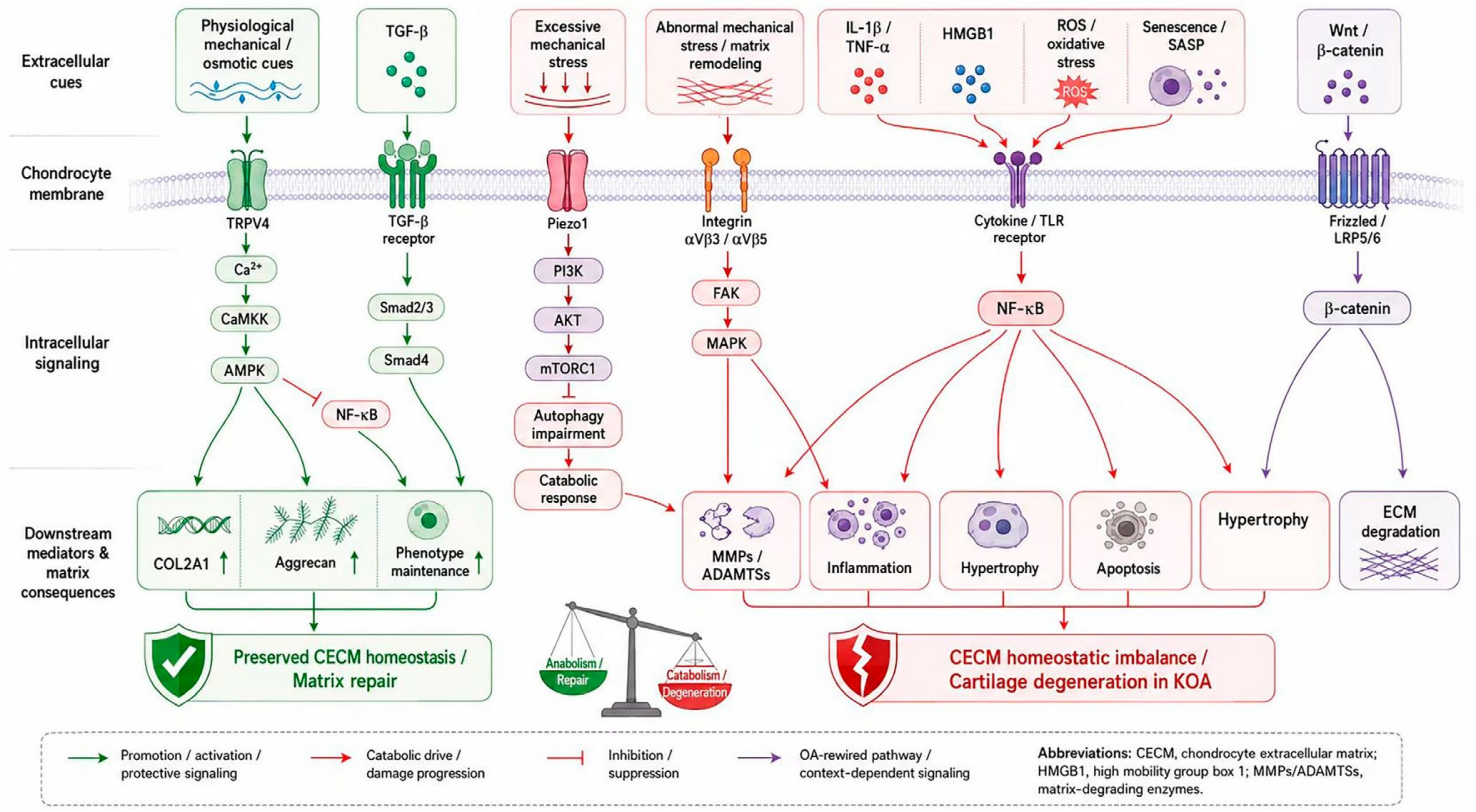
Core mechanisms underlying chondrocyte extracellular matrix homeostatic imbalance.

Beyond the mechanisms described above, sex represents an independent biological variable that profoundly influences CECM homeostasis through multiple molecular pathways. Transcriptomic analyses of human articular cartilage have revealed fundamental molecular distinctions between male and female articular cartilage: healthy female and male cartilage exhibit 36 differentially expressed genes, while in the osteoarthritic state, female cartilage displays 923 differentially expressed genes enriched in 15 signalling pathways compared with only 419 genes in 6 pathways in male cartilage [30]. Recent studies have further elucidated the hormonal regulation of CECM homeostasis. In a chemically induced menopausal mouse model and in postmenopausal human chondrocytes, Gilmer et al. [31] demonstrated that loss of 17β‐estradiol and progesterone in a chemically induced menopausal mouse model amplifies chondrocyte senescence and ECM disassembly, while hormone supplementation in postmenopausal human chondrocytes reduces senescence markers and increases chondrogenic markers. This underscores the necessity of incorporating sex as a critical biological variable in future mechanistic studies and therapeutic development for KOA.

Beyond the specific molecules and cellular processes discussed above, a complex network of key developmental and inflammatory signalling pathways orchestrates the transcriptional programs that govern CECM homeostasis. Among these, TGF‐β and Wnt/β‐catenin signalling play central and context‐dependent roles. In healthy cartilage, these pathways support matrix synthesis and maintain chondrocyte phenotype; however, in the osteoarthritic joint, their homeostatic functions are pathologically rewired. This shift creates a pro‐catabolic and pro‐hypertrophic environment that fundamentally drives ECM degradation and chondrocyte dysfunction. The intricate interactions and pathological shifts within these core signalling axes are integrated in Figure 1.

This diagram illustrates the intracellular signalling cascades that transduce diverse extracellular cues into discrete matrix metabolic outcomes in articular chondrocytes. The hierarchical organization moves from extracellular stimuli at the top, through membrane‐bound receptors and mechanosensors, to downstream transcriptional and enzymatic effectors that ultimately determine the balance between CECM synthesis and degradation. All abbreviated terms used in this figure are defined in the main text and Table S1.

At the cell membrane, chondrocytes integrate signals from multiple classes of receptors. Physiological mechanical and osmotic cues are primarily sensed by TRPV4, a mechanosensitive ion channel that mediates calcium influx. In the diagram, this pathway is shown to engage CaMKK, AMPK, and subsequently NF‐κB. While TRPV4 supports anabolic gene expression under physiologic loading, its downstream signalling in the osteoarthritic context reflects a pathologically rewired mechanotransduction axis that can shift toward catabolic outcomes, as dysfunction of the TRPV4–GSK3β axis impairs chondrocyte mechanosensing and indirectly facilitates matrix degeneration. TGF‐β signals through Smad2/3 and Smad4 to promote matrix synthesis and maintain chondrocyte phenotype under healthy conditions; however, in the OA joint, this pathway undergoes context‐dependent reprogramming that may redirect its output toward catabolic and hypertrophic responses. In contrast, excessive mechanical stress activates Piezo1, another mechanosensitive ion channel, which couples to the PI3K–AKT–mTORC1 cascade. This axis suppresses autophagic flux, reduces the synthesis of type II collagen and aggrecan, and promotes the expression of matrix‐degrading enzymes. Abnormal mechanical loading also activates integrins αVβ3 and αVβ5, which transduce mechanical signals through FAK and MAPK phosphorylation, ultimately upregulating inflammatory cytokines and matrix metalloproteinases. Pro‐inflammatory cytokines such as IL‐1β act through their cognate receptors to activate the NF‐κB pathway, which serves as a central hub for catabolic gene expression. Endogenous damage signals including HMGB1 engage TLR and RAGE receptors, amplifying inflammatory responses and further accelerating CECM degradation. Additionally, the Wnt/β‐catenin pathway is prominently featured as a pathologically rewired axis in OA that promotes chondrocyte hypertrophy and catabolic enzyme expression. Oxidative stress and endoplasmic reticulum stress activate stress‐responsive pathways that contribute to programmed cell death and matrix loss.

These converging signals ultimately dictate the net balance between anabolic and catabolic downstream mediators. On the anabolic side, enhanced production of COL2A1 (type II collagen) and aggrecan preserves matrix integrity and maintains the chondrocyte phenotype. On the catabolic side, upregulated MMPs and ADAMTSs directly degrade collagen and proteoglycan networks, while concurrent inflammatory responses, chondrocyte hypertrophy, and apoptosis further accelerate cartilage degeneration. The equilibrium between these opposing forces determines whether CECM homeostasis is preserved or shifted toward irreversible degradation and KOA progression.

It is noteworthy that several pathways depicted in this figure exhibit context‐dependent functionality. TGF‐β/Smad and TRPV4 signalling are protective under physiological conditions but may undergo pathological rewiring or functional impairment in the OA environment, shifting their net output toward catabolism. This contextual duality is a central theme in KOA pathogenesis and is elaborated in the main text. This figure serves as the molecular foundation for the integrated pathomechanism–therapeutic overview presented in Figure 2.

**FIGURE 2:**
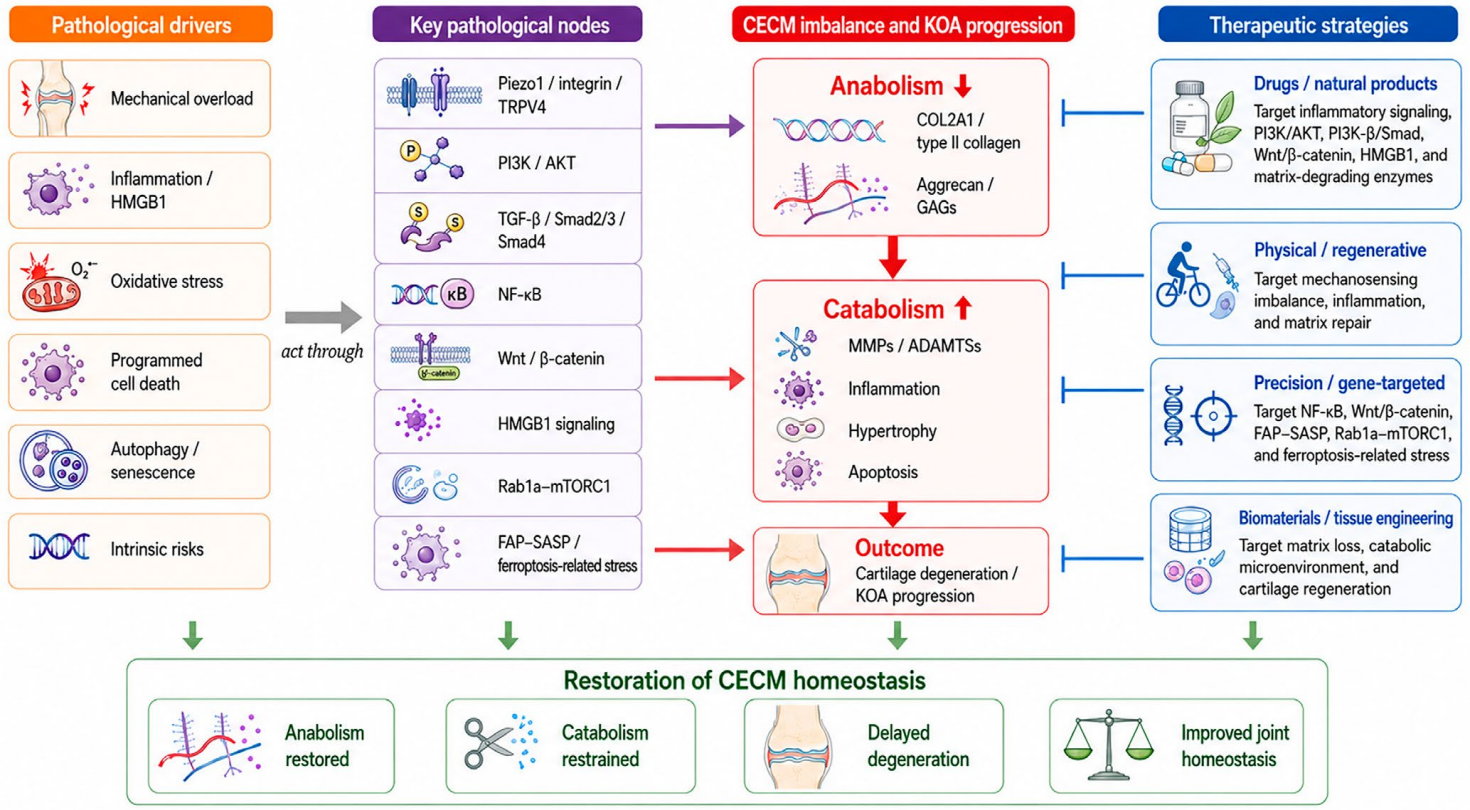
Schematic overview of KOA pathomechanisms and therapeutic strategies targeting CECM homeostasis.

## 4 Risk Factors for CECM Homeostasis

The homeostasis of the CECM is influenced by a multitude of factors, many of which are not only associated with the CECM itself but also increase the risk of developing KOA. Calcification of the CECM is a significant factor. Studies have found that Krüppel‐like factor 10 (Klf10) participates in regulating the calcification process of the CECM; knocking down Klf10 can mitigate CECM calcification, alleviate chondrocyte senescence, and improve the degeneration of articular cartilage [32]. Furthermore, aberrant expression of certain genes is also linked to alterations in the CECM and the onset of KOA. In studies on Kashin‐Beck disease patients and related animal models, exposure to T‐2 toxin can lead to the upregulation of the TSG‐6 gene in chondrocytes, thereby promoting CECM damage and degradation. This suggests that TSG‐6 may represent a potential risk factor for KOA [33].

Alterations in the mechanical properties of the CECM are also closely associated with the initiation and progression of KOA. In patients with KOA, changes in the composition and viscoelasticity of the CECM lead to an impaired perception of mechanical signals by chondrocytes [34]. Furthermore, dysfunction of the transient receptor potential vanilloid 4 (TRPV4)–glycogen synthase kinase 3β (GSK3β) pathway in KOA chondrocytes renders the cells unable to mount appropriate responses to changes in CECM viscoelasticity [21, 35]. This aberrant cellular mechanotransduction subsequently disrupts chondrocyte proliferation and phenotype, thereby promoting the development of KOA.

In addition to factors such as inflammation, oxidative stress, and mechanical loading, the release of endogenous alarmins represents another key biological process disrupting CECM homeostasis. High mobility group box 1 (HMGB1), a significant damage‐associated molecular pattern (DAMP), plays a dual role in OA pathology. In human OA cartilage and chondrogenic progenitor cells, Wagner et al. [36] found that HMGB1 expression is significantly higher in OA chondrocytes compared to healthy cartilage and relocates from the nucleus to the cytoplasm and extracellular space. Furthermore, the study confirmed that HMGB1 exerts a potent chemotactic effect on chondrogenic progenitor cells (CPCs), mediating their migration to injury sites via its receptors, the receptor for advanced glycation end products (RAGE) and Toll‐like receptor 4 (TLR4). This suggests that HMGB1 release in early OA may serve as an endogenous repair signal, attempting to recruit CPCs for tissue regeneration. However, chronic and excessive HMGB1 release can persistently activate inflammatory pathways, ultimately exacerbating CECM degradation. Consequently, the HMGB1‐RAGE/TLR4 signalling axis has emerged as a novel risk factor linking tissue damage, inflammation, and endogenous repair responses.

The progressive decline in the anabolic function of chondrocytes constitutes a core risk factor leading to CECM homeostatic imbalance, thereby driving KOA progression. It is particularly noteworthy that the low‐turnover nature of the cartilage collagen matrix renders it nearly incapable of effective regeneration following injury. In patients with late‐stage KOA, Jørgensen et al. [37] provided direct human evidence supporting this view. Using in vivo deuterium oxide (D_2_O) labelling to quantitatively analyse the collagen renewal rate in patients with late‐stage KOA, they found that despite differing regional mechanical environments (central weight‐bearing area vs. meniscus‐covered area), the fractional synthesis rate (FSR) of collagen was extremely low in both (0.027%/day and 0.022%/day, respectively). This indicates a severe failure in the capacity of chondrocytes to synthesize new collagen during the advanced stages of KOA. This profound metabolic dysfunction implies that even minor damage to the collagen network is likely irreversible, substantially compromising the structural integrity and mechanical function of the CECM. Consequently, the inherent deficit in collagen synthesis capacity, coupled with acquired metabolic inactivation, collectively forms a significant intrinsic risk factor for the initiation and progression of KOA. This insight also suggests that future KOA management strategies should place greater emphasis on activating the anabolic potential of chondrocytes to fundamentally reverse the loss of CECM.

Furthermore, external factors such as trauma and obesity can increase the risk of developing KOA by adversely affecting both the metabolism and mechanical properties of the CECM. Understanding these risk factors facilitates the early identification of high‐risk populations and enables the implementation of targeted preventive measures (Figure 2).

This figure provides a unified conceptual framework that integrates pathological drivers, core signalling nodes, matrix metabolic imbalance, and multi‐tiered therapeutic strategies. The left‐to‐right flow illustrates how diverse pathological stimuli converge onto key regulatory pathways, shifting the anabolic–catabolic balance of the CECM toward degradation, and ultimately driving cartilage degeneration and KOA progression. In parallel, therapeutic interventions are mapped to their respective targets to restore homeostatic equilibrium. All abbreviated terms used in this figure are defined in the main text and Table S1.

Several pathways depicted in this figure exhibit context‐dependent functions, meaning their biological outcomes vary according to the physiological or pathological milieu. For instance, TGF‐β/Smad signalling supports matrix synthesis and maintains the chondrocyte phenotype under healthy conditions; however, in the osteoarthritic joint, this pathway undergoes pathological rewiring and shifts toward promoting catabolic and hypertrophic responses. Similarly, TRPV4 mediates mechanosensing and promotes anabolic gene expression under physiologic mechanical loading, but its dysfunction in OA impairs the chondrocyte's ability to perceive changes in extracellular matrix viscoelasticity, indirectly facilitating degeneration. By contrast, other nodes shown here, including Piezo1/integrin, NF‐κB, Wnt/β‐catenin (pathologically rewired in OA), HMGB1 signalling, Rab1a–mTORC1, and ferroptosis‐related stress, predominantly drive catabolic cascades, upregulate matrix‐degrading enzymes, and promote cellular dysfunction in the OA context. Based on these mechanistic insights, therapeutic strategies are categorized into four major modalities: pharmacological agents (including natural products), physical and regenerative therapies, precision and gene‐targeted interventions, and biomaterial‐based approaches. Each category acts on distinct pathological nodes to restore anabolic activity, restrain catabolic processes, and ultimately delay the progression of KOA.

## 5 Application of CECM‐Associated Biomarkers in KOA Diagnosis and Prognostic Evaluation

Imaging techniques play a crucial role in the clinical diagnosis of KOA. However, each modality presents distinct advantages and limitations. Although conventional radiography is widely used, it exhibits limited sensitivity for detecting early‐stage cartilage damage. Magnetic resonance imaging (MRI), although highly valuable for assessing articular cartilage pathology, is constrained by its relatively high cost and prolonged scanning time, limiting its broad clinical application. In contrast, computed tomography (CT) offers superior evaluation of osseous structures but remains suboptimal for detecting chondral lesions. Consequently, biomarkers capable of directly reflecting the dynamic metabolic status of the CECM provide an essential complement for early diagnosis, disease monitoring, and treatment efficacy evaluation in KOA.

### 5.1 Body Fluid Biomarkers

Degradation products of the CECM, such as cartilage oligomeric matrix protein (COMP), are significantly elevated in the serum and synovial fluid of KOA patients and correlate with disease severity [38, 39]. Measurement of serum COMP levels aids in the diagnosis and disease assessment of KOA. Meta‐analyses have substantiated its diagnostic utility [40], demonstrating that serum COMP levels can effectively distinguish KOA patients from healthy controls, while elevated baseline levels are consistently associated with a significantly increased risk of radiographic progression over 2–3 years, supporting its prognostic relevance. Type II collagen degradation products, such as C‐terminal cross‐linking telopeptide of type II collagen (C2C, also known as uCTX‐II), also show a positive correlation with the extent of joint tissue damage and can be used to evaluate pathological progression [41]. Clinical cohorts have established uCTX‐II as a specific indicator of rapid structural deterioration, with elevated levels conferring a two‐ to three‐fold increased odds of disease progression, although its modest sensitivity for early‐stage detection highlights the need for complementary markers [42].

Furthermore, non‐coding RNAs involved in regulating CECM metabolism show considerable potential. For instance, miR‐449a promotes CECM degradation by targeting growth differentiation factor 5 (GDF5). Detecting miR‐449a expression levels can provide insights into KOA pathogenesis and disease progression [43], and preliminary discriminative analyses have demonstrated encouraging performance (AUC ~0.817) for differentiating KOA grades; however, independent external validation in diverse populations remains a prerequisite for clinical translation [44]. On the other hand, certain long non‐coding RNAs (lncRNAs) influence chondrocyte apoptosis and CECM degradation by modulating relevant signalling pathways, showing promise as novel diagnostic and therapeutic targets for KOA [45]. Liu et al. [46] identified a substantial number of differentially expressed lncRNAs (e.g., MSTRG.95856) in KOA cartilage through high‐throughput sequencing. Their target genes were significantly enriched in classic KOA pathways such as the PI3K‐Akt signalling and ECM‐receptor interaction, providing a valuable data resource for discovering new diagnostic markers and intervention targets.

### 5.2 Imaging Biomarkers

In addition to the molecular biomarkers in body fluids discussed above, quantitative biomarkers derived from medical imaging have emerged as powerful tools for assessing CECM metabolism and joint structural changes, owing to their non‐invasive nature, reproducibility, and precise localization. Particularly, quantitative magnetic resonance imaging (qMRI) techniques enable the non‐invasive evaluation of the biochemical composition of articular cartilage and the microarchitecture of subchondral bone, offering new perspectives for KOA diagnosis and management [47]. Among these, T2 mapping sequences measure tissue T2 relaxation times to reflect the integrity of the collagen fibre network and water content within cartilage. Elevated T2 values indicate collagen network disruption and/or increased hydration, serving as a sensitive indicator of early CECM degeneration. A systematic meta‐analysis has confirmed that T2 relaxometry significantly discriminates between mild OA and healthy controls [48], with this discriminative ability further validated in a large population‐based cohort [49]. Beyond its diagnostic utility, baseline T2 mapping also offers prognostic value, as T2 signal abnormalities have been shown to predict future structural cartilage deterioration [50]. Meanwhile, trabecular bone analysis based on high‐resolution MRI can quantitatively assess the microarchitecture of subchondral bone (e.g., apparent bone fraction, trabecular number, and separation). The remodelling and sclerosis of subchondral bone represent critical processes in KOA progression and are closely associated with cartilage degeneration [51]. Gersing et al. [52], in a prospective study, validated the prognostic predictive value of such imaging biomarkers. In patients undergoing cartilage repair surgery, global cartilage T2 values and subchondral trabecular parameters measured as early as 3 months postoperatively effectively predicted superior graft morphology and less severe whole‐joint degenerative changes at the 2‐year follow‐up. This demonstrates that quantitative MRI parameters can evolve from diagnostic tools into predictive prognostic biomarkers. Subchondral trabecular parameters demonstrate diagnostic performance comparable to that of cartilage‐specific sequences. Critically, the integration of these imaging metrics with fluid biomarkers has been shown to yield synergistic predictive value for mid‐term clinical outcomes [53]. A growing body of evidence supports that such a dual‐modal approach can effectively bridge molecular perturbations with their structural consequences [54], thereby enhancing the precision of KOA evaluation and offering a more comprehensive framework for disease stratification and prognostic assessment [55, 56].

## 6 Therapeutic Strategies Targeting CECM

The therapeutic strategies discussed below encompass evidence ranging from preclinical investigations to early‐phase clinical trials. While substantial progress has been made in animal models and in vitro systems, clinical validation remains limited for many approaches, and readers should interpret the findings with appropriate consideration of their translational stage.

### 6.1 Pharmacological Treatment

Pharmacological treatment for KOA aims to alleviate pain, improve joint function, and delay disease progression. Research into drugs targeting symptom relief and disease modification continues to advance.

Conventional disease‐modifying agents, such as chondroitin sulphate (CS), serve as both key components of the cartilage matrix and symptomatic slow‐acting drugs in osteoarthritis (SYSADOA). Debates surrounding its efficacy are often linked to its source and purity. In recent years, high‐purity, microbially‐derived CS (MCS) produced via biotechnology has demonstrated superior potential. Sevimli et al. [57] confirmed in an experimental rabbit OA model that MCS sourced from *Escherichia coli* was significantly more effective than traditional animal‐derived CS in improving articular surface structure and reducing cartilage erosion and cell necrosis. This not only validates the mechanism by which CS acts—by modulating CECM metabolic balance, suppressing inflammation, and inhibiting degrading enzyme activity—but also provides a novel pathway for obtaining new, high‐purity, lower‐risk CS formulations.

Natural products and active components from Traditional Chinese Medicine (TCM), valued for their multi‐target and multi‐pathway regulatory profiles, represent an important source for interventions targeting CECM metabolism. Research in this area has evolved from crude extracts to active monomers and complex formulations, with mechanisms becoming increasingly elucidated.

In the realm of active monomers, several structurally defined natural compounds regulate CECM homeostasis by acting on specific signalling nodes. The flavonoid casticin inhibits the activation of the PI3K/AKT/HIF‐1α pathway, thereby downregulating the expression of inflammatory mediators (IL‐1β, TNF‐α, iNOS, COX‐2) and apoptosis‐related proteins (Bax/Bcl‐2, caspase‐3/8), while simultaneously reversing ECM metabolic imbalance (promoting COL2A1 and ACAN synthesis; inhibiting MMP‐9, MMP‐13, ADAMTS4/5), exerting protective effects across multiple levels [58]. Polysaccharide components, such as *Polygonatum sibiricum* polysaccharide (PSP), were demonstrated for the first time to target TLR2 as a key receptor. By inhibiting the activation of the TLR2/NF‐κB axis, PSP effectively suppresses IL‐1β‐induced release of inflammatory factors and upregulates COL2A1 expression [59]. The terpenoid curcumenol extends its mechanism to the level of epigenetic and metabolic regulation. It directly targets the histone demethylase KDM6B, inhibits H3K27me3 modification, and downregulates succinic acid levels, thereby mitigating inflammatory stress and promoting collagen synthesis [60]. The coumarin compound osthole focuses on autophagy regulation. It significantly enhances autophagic flux by activating the AMPK/ULK1 signalling pathway, inhibiting IL‐1β‐induced chondrocyte apoptosis and ECM degradation [61].

At the level of TCM effective fractions and compound formulations, their multi‐component nature confers an overall advantage of multi‐link, synergistic intervention. The effective fraction drug derived from white peony, Total Glucosides of White Peony Capsule (TGP), has been revealed to possess a dual ‘cartilage‐bone’ protective mechanism. On one hand, it inhibits the NF‐κB pathway to reduce the expression of inflammatory factors and matrix‐degrading enzymes (MMP3, MMP13, ADAMTS5); on the other hand, it modulates the OPG/RANKL/RANK system, ameliorating abnormal subchondral bone remodelling [62]. Conversely, Quan Du Zhong Capsule was discovered via spatial transcriptomics to specifically act on the superficial and middle zones of articular cartilage. By inhibiting the consecutive signalling axis TXNIP‐NLRP3‐BMPR2‐FN1‐MMP2, it effectively alleviates local oxidative stress and inflammatory responses, thereby suppressing the activity of enzymes like MMP2 and reducing the loss of ECM components [63]. The classic compound formulation Duhuo Jisheng Decoction demonstrates versatility in regulating cell death modalities. It can downregulate the PI3K/Akt/mTOR pathway to suppress the NLRP3/caspase‐1/GSDMD axis, thereby reducing pyroptosis and the subsequent release of IL‐1β, IL‐18, and MMP13 [64]. Alternatively, it can upregulate PTEN expression to inhibit the Akt/mTOR pathway, consequently enhancing autophagic activity and indirectly suppress MMP‐13 expression by reducing the availability of its zinc ion cofactor [65].

Furthermore, novel agents with defined compositions, developed based on traditional Chinese medicine (TCM) knowledge, are continuously emerging. For instance, three bioactive deer antler peptides (TSKYR, TSK, YR) were identified from the classical formulation Guilu Erxian Jiao. In a collagenase‐induced rat KOA model, intra‐articular injection of these peptides demonstrated chondroprotective effects comparable or even superior to hyaluronic acid, effectively reducing matrix loss and better preserving chondrocytes and proteoglycans. This provides a successful paradigm for developing new therapeutic peptide drugs [66].

It is noteworthy that the mechanisms of action of natural products can be diverse and not exclusively target classical inflammatory or CECM metabolic pathways. For example, in a secondary analysis of a randomized controlled trial in KOA patients [67], it was indicated that while *Curcuma longa* extract significantly alleviated patient pain, no significant improvements were observed in serum inflammatory markers (TNF‐α, IL‐6, hsCRP), MMP‐3 concentration, or CE‐MRI‐assessed synovitis. This suggests that its analgesic effect may originate from neuromodulation or other non‐direct anti‐inflammatory pathways.

### 6.2 Physical and Regenerative Therapy

Physical intervention and regenerative medicine strategies aim to modulate the intra‐articular microenvironment, thereby promoting CECM synthesis and cartilage self‐repair, offering novel avenues for KOA treatment.

#### 6.2.1 Physical Stimulation and Electroacupuncture Therapy

Physical therapy not only alleviates symptoms but may also influence CECM homeostasis through immunomodulation. Yan et al. [68] applied a modified tug‐of‐war acupuncture technique (BHZF) to treat a rabbit KOA model, finding that it significantly relieved pain, improved cartilage structure, and regulated the polarization direction of intra‐articular macrophages by suppressing pro‐inflammatory M1 polarization while promoting anti‐inflammatory M2 polarization. This consequently reduced levels of pro‐inflammatory factors such as IL‐1β and TNF‐α while elevating levels of anti‐inflammatory factors including IL‐4 and IL‐10, thereby revealing the potential mechanism of acupuncture in treating KOA from the perspective of immunomodulation within the joint microenvironment.

#### 6.2.2 Cell Therapy and Stem Cell Applications

Stem cell transplantation represents a core strategy in regenerative medicine, primarily regulating chondrocyte function and CECM metabolism through paracrine effects. Regenerative medicine offers new hope for KOA treatment, wherein the CECM itself holds significant application potential. As a vital component of regenerative medicine, stem cell therapy involves the intra‐articular injection of stem cells, such as mesenchymal stem cells (MSCs), to promote CECM synthesis and cartilage regeneration. However, further research is required to determine the optimal treatment protocols and long‐term efficacy. Long‐term follow‐up studies have found that some patients may experience disease recurrence after a period post‐stem cell therapy, with the underlying mechanisms remaining unclear. Noorwali et al. [69], via intra‐articular injection of rat bone marrow‐derived MSCs (rBM‐MSCs) in a rat KOA model, demonstrated a reduction in multiple serum biomarker levels (HA, NTX‐I, COMP, MMP‐3, IL‐6) and promotion of cartilage structural repair. Bioinformatics analysis suggested these effects were associated with the modulation of Wnt/β‐catenin and TGF‐β signalling pathways. Zhang et al. [70] confirmed that allogeneic adipose‐derived MSCs (ADSCs) promoted the redifferentiation of endogenous chondrocytes (upregulating Col2a1, Aggrecan, Sox9; downregulating Col1a1, Col10a1, Runx2) via paracrine actions in a rabbit KOA model, enhanced their proliferation and migration, suppressed MMP13 expression, thereby delaying cartilage degeneration without eliciting significant immune rejection. Furthermore, Chen et al. [71] found that Nanofat, a minimally processed adipose‐derived cell mixture requiring no in vitro expansion, demonstrated efficacy comparable to ADSCs in both preclinical rat OA models and in clinical patients. It alleviated pain, improved cartilage structure, and regulated the balance of chondrocyte anabolism and catabolism via paracrine mechanisms, showcasing clinical utility as an immediate‐use therapeutic.

In recent years, various biomaterial carriers have been developed to enhance stem cell retention and survival within the joint cavity. Chen et al. [72] utilized gelatin microspheres (GMs) to load Wharton's jelly‐derived mesenchymal stem cells (WJMSCs), demonstrating that GMs not only maintained cell viability and promoted proliferation and chondrogenic differentiation (upregulating Col2a1 and Sox9) but also significantly suppressed the expression of the hypertrophy marker Col10a1. In a mouse KOA model induced by anterior cruciate ligament transection (ACLT)/destabilization of the medial meniscus (DMM), transplantation of the WJMSCs‐GMs complexes markedly promoted cartilage matrix synthesis, reduced Mankin scores, and prolonged intra‐articular cell retention. Further mechanistic investigation suggested that the complex likely modulates synovial macrophage polarization toward the M2 phenotype, thereby improving the local inflammatory microenvironment and creating favourable conditions for CECM repair. This study provides new experimental evidence for stem cell–biomaterial combination therapy, highlighting the crucial value of functional biomaterials in enhancing the efficacy and durability of stem cell‐based treatments.

Beyond cell transplantation strategies, the direct utilization of a patient's own cartilage tissue for repair represents an emerging regenerative medicine approach. Tsuyuguchi et al. [73] proposed an optimized protocol: mincing cartilage tissue directly into tiny fragments (Minced Cartilage, MC) and embedding them within a collagen gel, thereby maximally preserving the interaction between chondrocytes and their native CECM. Compared to traditional autologous chondrocyte implantation (ACI), which requires enzymatic digestion and isolation, the MC strategy significantly enhanced chondrocyte migration and proliferation capabilities, synthesized more glycosaminoglycans (GAG), and achieved superior repair outcomes using only one‐quarter of the cartilage harvest required by the conventional method, substantially reducing the risk of donor‐site morbidity. This approach effectively maintains chondrocyte function even within a degenerative environment, thus providing a novel perspective for developing simpler and more efficient one‐step cartilage repair procedures.

#### 6.2.3 Bioactive Factor Therapy

Zhuo et al. [74] demonstrated that platelet‐rich plasma (PRP) alleviates inflammation, promotes chondrocyte proliferation, suppresses apoptosis, and reverses ECM metabolic imbalance in a rat KOA model by inhibiting the NF‐κB key subunit p65. The study further proposed that combining PRP with p65‐targeted gene therapy could potentially enhance therapeutic efficacy. In a separate study, Lu et al. [75] found that the combined application of a PEDF‐derived peptide (29‐mer) with hyaluronic acid (HA) activates the PEDFR/STAT3 signalling pathway, thereby enhancing the proliferation and chondrogenic differentiation capacity of bone marrow‐derived mesenchymal stem cells (BM‐MSCs)—evidenced by upregulated expression of Sox9, aggrecan, and COL2A1. In a rat OA model, this combination promoted chondrocyte regeneration and increased the deposition of type II collagen and proteoglycans.

### 6.3 Gene and Targeted Therapy

Gene and targeted therapies aim to provide novel treatment strategies for KOA by precisely targeting key genes or signalling pathways involved in CECM metabolism.

#### 6.3.1 Gene Editing and Stem Cell Potentiation

The advancement of gene editing technology provides novel avenues for enhancing the therapeutic efficacy of stem cells. Zhang et al. [76] demonstrated that ribosomal S6 kinase RSK3 (encoded by the Rps6ka2 gene) is crucial for maintaining the regenerative capacity of chondrocytes. Utilizing CRISPR/Cas9 technology to generate induced pluripotent stem cell‐derived MSCs (iMSCs) with Rps6ka2 knockout significantly impaired their proliferation and chondrogenic differentiation capabilities. In a mouse KOA model induced by DMM, transplantation of wild‐type iMSCs promoted type II collagen synthesis, suppressed MMP‐13 expression, and delayed cartilage degeneration. Conversely, iMSCs with the gene knockout exhibited markedly reduced therapeutic efficacy, highlighting the potential of optimizing CECM synthesis through gene editing.

#### 6.3.2 Epigenetic Regulation

Targeting epigenetic regulators to reverse chondrocyte senescence and metabolic imbalance has become a research focus. Collins et al. [29] discovered that SIRT6 activity is significantly reduced in senescent chondrocytes, and its deficiency suppresses the IGF‐1/Akt signalling pathway, consequently decreasing the synthesis of key CECM components such as COL2A1. Pharmacological activation of SIRT6 with the small‐molecule agonist MDL‐800 promoted Akt phosphorylation and activated anabolic signalling, indicating that targeting SIRT6 holds promise for promoting CECM synthesis and delaying OA progression.

#### 6.3.3 RNA Interference and Targeted Delivery

Targeted therapies based on RNA interference (RNAi) show promising application prospects. Zhao et al. [28] developed lipid nanoparticles loaded with FAP siRNA (LNP@FAP siRNA). This delivery system exhibits excellent biocompatibility and sustained‐release capability within the joint cavity. In a rat ACLT model, intra‐articular injection significantly reduced FAP expression in cartilage, inhibited cellular senescence, and alleviated cartilage degeneration and synovitis, providing both a novel therapeutic target and an advanced delivery strategy for OA gene therapy.

#### 6.3.4 Engineered Cells and Immune Microenvironment Modulation

Engineered cell therapy promotes CECM repair by modulating the joint immune microenvironment. TissueGene‐C (TG‐C) is a cell‐based gene therapy comprising allogeneic human chondrocytes and genetically engineered cells expressing TGF‐β1. In in vitro studies using a THP‐1 macrophage polarization model, Tran et al. [77] revealed that the engineered cells continuously secrete TGF‐β1, which stimulates chondrocytes to produce prostaglandin E2 (PGE2). PGE2, in turn, drives macrophage polarization toward the M2 phenotype via the EP4 receptor on macrophages, upregulating anti‐inflammatory factors IL‐10 and IL‐1ra while suppressing TNF‐α secretion, thereby remodelling the immune microenvironment and creating favourable conditions for CECM repair.

#### 6.3.5 Targeting Signalling Pathways and Membrane Receptors

Notable progress has been made in biologics targeting specific signalling pathways or cell membrane receptors. In a Phase I clinical trial, Gerwin et al. [78] reported on LNA043, a derived protein targeting integrin α5β1, which promotes the differentiation of human mesenchymal stem cells into chondrocytes, increases the synthesis of PRG4 and type II collagen, and induces the secretion of Dickkopf‐1 (DKK1), an inhibitor of the WNT pathway. A Phase I clinical trial demonstrated that a single intra‐articular injection significantly reversed OA‐related gene expression patterns, downregulating pathological mediators such as FN1 and SPP1 while upregulating matrix components including COL9A1 and MATN4. These findings demonstrate its strong potential to regulate the balance between CECM synthesis and degradation, positioning LNA043 as a promising disease‐modifying osteoarthritis drug (DMOAD) candidate.

Additionally, supplementing protective factors like SELM represents another therapeutic strategy. Zhao et al. [25] showed that activating SELM expression alleviates endoplasmic reticulum stress and ferroptosis by inhibiting the PERK/ATF4/CHAC1 pathway, thereby protecting CECM homeostasis through multiple mechanisms.

### 6.4 Tissue Engineering and Material‐Based Strategies

Tissue engineering and biomaterial strategies represent a pivotal direction for promoting CECM regeneration. This is achieved by constructing functionalized scaffolds or delivery systems that mimic the microenvironment of the native CECM, thereby providing physical support for cartilage repair and actively regulating biological processes.

#### 6.4.1 Multifunctional Hydrogel Scaffolds

Injectable hydrogels are widely employed to fill defects and promote host integration due to their excellent biocompatibility and formability. Wang et al. [27] developed a gelatin‐chitosan composite hydrogel incorporating Mo_2_Ti_2_C_3_ MXene. This material demonstrated the ability to scavenge reactive oxygen species (ROS) under near‐infrared (NIR) light irradiation, modulate macrophage polarization toward the M2 phenotype (reducing IL‐1β and IL‐6 while elevating TGF‐β and IL‐10), and significantly promote the synthesis of aggrecan and type II collagen. In a mouse OA model, it effectively delayed cartilage degeneration.

In a separate study, Zhu et al. [79] designed a dual‐crosslinked hydrogel (GM/HN) with robust wet adhesion. The scaffold provides mechanical support through photocrosslinked methacrylated gelatin (GelMA), while NB‐modified hyaluronic acid (HANB) covalently bonds to tissue via Schiff base reactions. Increasing the HANB proportion synergistically enhanced both its compressive modulus (~29 kPa) and adhesive strength (burst pressure ~40 kPa). After 6 weeks of implantation in a rabbit cartilage defect model, the regenerated cartilage tissue in the defect area was rich in glycosaminoglycans (GAG) and type II collagen, showing excellent integration with the surrounding native tissue.

#### 6.4.2 Sustained‐Release and Targeted Delivery Systems

Biomaterial‐based sustained‐release systems enable controlled delivery of bioactive factors, significantly enhancing treatment durability. Zhu et al. [80] developed a dual‐layer HA/Wnt16@MSN system by encapsulating Wnt16‐loaded mesoporous silica nanoparticles within a hyaluronic acid (HA) hydrogel, achieving both rapid initial release and prolonged sustained release. In a rat temporomandibular joint osteoarthritis (TMJOA) model, this system promoted cartilage matrix synthesis while simultaneously suppressing inflammation and osteoclast activity. In a similar approach, Li et al. [81] co‐encapsulated mesenchymal stem cell‐derived extracellular vesicles (MSC‐EVs) with icariin (ICA) in a sodium alginate‐hyaluronic acid (SA‐HA) hydrogel to construct an injectable sustained‐release system. In a rat cartilage defect model, this combined system synergistically upregulated COL2A1 and ACAN expression while inhibiting MMP13 and ADAMTS5, resulting in nearly complete cartilage regeneration within 8 weeks. Innovatively, Wei et al. [82] developed cationic micellar nanoparticles loaded with a phospholipase A_2_ inhibitor (sPLA_2_i‐NPs). The positive surface charge of these nanoparticles enables deep tissue penetration and prolonged joint retention through electrostatic interactions with cartilage glycosaminoglycans (GAGs). In osteoarthritis models, sPLA_2_i‐NPs effectively inhibited sPLA_2_ activity and the NF‐κB pathway, thereby alleviating cartilage degradation and synovitis.

#### 6.4.3 Biomimetic Scaffolds and Structural Design

Biomimetic design optimizes repair outcomes by mimicking the hierarchical structure and chemical composition of native cartilage. Wang et al. [83] designed a bilayered biomimetic cartilage scaffold: the superficial layer (composed of collagen/chitosan/sodium hyaluronate) was loaded with KGN microspheres to induce chondrogenic differentiation, while the transitional layer (composed of collagen/chitosan/silk fibroin) incorporated aligned microtubule arrays and TGF‐β1 nanoparticles to promote cell migration and proliferation. In a rabbit full‐thickness cartilage defect model, implantation for 16 weeks resulted in complete defect filling, with the neo‐tissue exhibiting well‐arranged chondrocytes and abundant proteoglycans. The repair outcome was significantly superior to that achieved with a single‐layer scaffold.

Meanwhile, in a prospective multicenter clinical study of 86 patients with knee OA followed for 2 years, Kon et al. [84] evaluated an aragonite‐based biphasic scaffold (Agili‐C), with significant improvements observed in both KOOS and IKDC scores. MRI assessments revealed a defect fill rate of 78.7%, and histology confirmed the formation of type II collagen‐positive hyaline‐like cartilage.

#### 6.4.4 Integrated Therapeutic Strategies

Integrated ‘material‐drug‐cell’ combination strategies demonstrate substantial potential. For instance, Hossain et al. [85] constructed a hydrogel‐based sustained‐release system loaded with ginsenoside Rb1. In a rabbit KOA model, this system inhibited the PI3K/Akt, p38/MAPK, and NF‐κB pathways, downregulated MMP expression, reduced ROS generation and apoptosis, promoted collagen synthesis, and significantly restored proteoglycan and type II collagen content in the cartilage structure. Separately, Palma et al. [86] developed a mechanically active organ‐on‐a‐chip platform (uBeat MultiCompress) capable of simulating OA‐relevant mechanical loading in vitro. Using this platform, they evaluated the sustained‐release formulation SYN321 (a diclofenac‐hyaluronate conjugate), confirming its ability to downregulate inflammatory factors and MMP13 expression while promoting aggrecan deposition. This provides a novel platform for screening CECM intervention strategies under pathologically relevant mechanical conditions.

### 6.5 Summary

Maintaining CECM homeostasis represents a core strategy for both preventing and treating KOA. This section has reviewed various therapeutic strategies targeting the CECM and their recent advancements. Pharmacological treatment has evolved from traditional symptom relief toward multi‐targeted and refined modulation. High‐purity microbial‐derived chondroitin sulphate, numerous natural active monomers with clearly defined mechanisms (e.g., Casticin, Curcumenol, Osthole), TCM effective fractions (e.g., Total Glucosides of Paeony Capsule, Quan Du Zhong Capsule), and compound formulations (e.g., Duhuo Jisheng Decoction) synergistically protect the CECM through anti‐inflammatory, antioxidant, and regulation of autophagy and cell death pathways (Table 1).

**TABLE 1:** Pharmacological agents targeting CECM homeostasis in KOA: Mechanisms and evidence levels.

Category	Agent	Key mechanism (summarized)	Evidence level
Traditional disease‐modifying drugs	High‐purity microbial‐derived chondroitin sulphate (MCS)	Modulates ECM metabolic balance; suppresses inflammation and degrading enzyme activity	Preclinical (rabbit OA model) [57]
Natural active monomers	Casticin (flavonoid)	Inhibits PI3K/AKT/HIF‐1α; suppresses inflammatory mediators and apoptosis; reverses ECM imbalance (promotes COL2A1/ACAN, inhibits MMPs/ADAMTSs)	Preclinical (rat OA model) [58]
	*Polygonatum sibiricum* polysaccharide (PSP)	Targets TLR2; inhibits TLR2/NF‐κB axis; suppresses IL‐1β‐induced inflammation; upregulates COL2A1	Preclinical (in vitro+in vivo) [59]
	Curcumenol (terpenoid)	Targets histone demethylase KDM6B; inhibits H3K27me3; downregulates succinic acid	Preclinical (cellular + animal) [60]
	Osthole (coumarin)	Activates AMPK/ULK1; enhances autophagic flux; inhibits IL‐1β‐induced chondrocyte apoptosis and ECM degradation	Preclinical (in vitro + in vivo) [61]
TCM effective fractions & compound formulations	Total Glucosides of Paeony Capsule (TGP)	Dual cartilage‐bone protection: (1) inhibits NF‐κB, reduces MMPs/ADAMTSs; (2) modulates OPG/RANKL/RANK, ameliorates subchondral bone remodelling	Preclinical (rat OA model) [62]
	Quan Du Zhong Capsule	Acts on superficial/middle cartilage zones (spatial transcriptomics); inhibits TXNIP‐NLRP3‐BMPR2‐FN1‐MMP2 axis; alleviates oxidative stress and inflammation	Preclinical (spatial transcriptomics + animal model) [63]
	Duhuo Jisheng Decoction	Dual regulation: (1) inhibits PI3K/Akt/mTOR/NLRP3/caspase‐1 axis → reduces pyroptosis; (2) upregulates PTEN → inhibits Akt/mTOR → enhances autophagy	Preclinical (rabbit/rat OA models) [64, 65]
Novel TCM‐derived agents	Deer antler peptides (TSKYR, TSK, YR)	Chondroprotective; reduces matrix loss; preserves chondrocytes and proteoglycans	Preclinical (rat KOA model) [66]
Special case	*Curcuma longa* extract	Analgesic effect likely via neuromodulation, not direct anti‐inflammatory or CECM pathways (no significant changes in serum inflammatory markers or synovitis by CE‐MRI)	Clinical (RCT secondary analysis) [67]

Physical and regenerative therapies have moved beyond mere structural repair to focus on modulating the intra‐articular microenvironment. The efficacy of stem cell therapies (e.g., MSCs, ADSCs, Nanofat) primarily stems from their paracrine effects, while the incorporation of biomaterial carriers significantly enhances cell retention and survival. Minimally invasive techniques based on autologous cartilage fragments maximize the preservation of cell‐native CECM interactions, demonstrating unique advantages.

Gene and targeted therapies are progressing toward precision and personalization. Approaches such as gene editing (e.g., CRISPR/Cas9 targeting Rps6ka2), epigenetic regulation (e.g., SIRT6 activation), RNA interference (e.g., FAP‐targeting siRNA), and engineered cell therapies (e.g., TG‐C) aim to correct CECM metabolic imbalances at their root.

Tissue engineering and biomaterial strategies utilize functionalized biomaterials (e.g., MXene hydrogels, biomimetic bilayered scaffolds, sustained‐release nano‐systems) to mimic the physicochemical and biological microenvironment of the native CECM. These strategies not only provide physical support for cells but also actively regulate the immune microenvironment and promote matrix synthesis, accomplishing a transition from ‘passive filling’ to ‘active regeneration’ (Table 2).

**TABLE 2:** Multimodal therapeutic strategies for KOA: Mechanisms, targets and translational stage.

Category	Strategy	Mechanism/target (summarized)	Evidence level
Physical stimulation	Modified tug‐of‐war acupuncture (BHZF)	Promotes M2 macrophage polarization; reduces IL‐1β/TNF‐α; increases IL‐4/IL‐10	Preclinical (rabbit KOA model) [68]
Stem cell therapy	rBM‐MSCs	Modulates Wnt/β‐catenin and TGF‐β; reduces serum biomarkers (COMP, MMP‐3, IL‐6)	Preclinical (rat KOA model) [69]
	Allogeneic ADSCs	Paracrine effects; promotes chondrocyte redifferentiation (Col2a1/aggrecan/Sox9 ↑; Col1a1/Col10a1/Runx2 ↓); inhibits MMP13	Preclinical (rabbit KOA model) [70]
	Nanofat (adipose‐derived cell mixture)	Paracrine regulation of anabolic/catabolic balance; no in vitro expansion required	Preclinical + clinical (rat models + patients) [71]
	WJMSCs + gelatin microspheres (GMs)	GMs maintain viability and promote chondrogenesis (Col2a1/Sox9 ↑, Col10a1 ↓); modulates M2 macrophage polarization	Preclinical (mouse KOA model) [72]
Tissue‐based repair	Minced cartilage (MC) + collagen gel	Preserves chondrocyte‐CECM interaction; enhances migration/proliferation; increases GAG synthesis; reduces donor site morbidity	Preclinical (in vitro) [73]
Bioactive factor therapy	Platelet‐rich plasma (PRP)	Inhibits NF‐κB p65; promotes proliferation, inhibits apoptosis; reverses ECM imbalance	Preclinical (rat KOA model) [74]
	PEDF‐derived peptide (29‐mer) + HA	Activates PEDFR/STAT3; enhances BM‐MSC chondrogenesis (Sox9/aggrecan/COL2A1 ↑)	Preclinical (rat OA model) [75]
Gene editing	CRISPR/Cas9 (Rps6ka2 targeting in iMSCs)	RSK3 maintains chondrocyte regenerative capacity; Rps6ka2 knockout reduces therapeutic efficacy	Preclinical (in vitro + mouse model) [76]
Epigenetic regulation	SIRT6 agonist (MDL‐800)	Activates SIRT6; promotes Akt phosphorylation and anabolic signalling; reverses senescence	Preclinical (mouse model + in vitro) [29]
RNA interference	FAP siRNA (LNP@FAP siRNA)	Reduces FAP expression in cartilage; inhibits senescence; alleviates degeneration and synovitis	Preclinical (rat ACLT model) [28]
Engineered cell therapy	TissueGene‐C (TG‐C)	Engineered TGF‐β1 secretion → PGE2 production → M2 macrophage polarization via EP4; upregulates IL‐10/IL‐1ra, inhibits TNF‐α	Preclinical (in vitro) [77]
Targeted biologics	LNA043 (anti‐integrin α5β1)	Promotes MSC chondrogenesis; increases PRG4/COL2A1; induces DKK1 (WNT inhibitor); reverses OA gene expression	Phase I clinical trial [78]
	SELM supplementation	Inhibits PERK/ATF4/CHAC1; alleviates ER stress and ferroptosis	Preclinical (human OA samples + mouse model) [25]
Hydrogel scaffolds	Mo_2_Ti_2_C_3_ MXene hydrogel	Scavenges ROS under NIR; promotes M2 polarization; delays cartilage degeneration	Preclinical (mouse OA model) [27]
	GelMA/HANB dual‐crosslinked hydrogel	Provides mechanical support; covalent tissue bonding; promotes integration with native tissue	Preclinical (rabbit cartilage defect model) [79]
Sustained‐release systems	HA/Wnt16@MSN system	Dual‐layer release; promotes matrix synthesis; inhibits inflammation and osteoclast activity	Preclinical (rat TMJOA model) [80]
	MSC‐EVs + icariin (ICA) in SA‐HA hydrogel	Synergistically upregulates COL2A1/ACAN; inhibits MMP13/ADAMTS5	Preclinical (rat cartilage defect model) [81]
	sPLA_2_i‐loaded micellar NPs (sPLA_2_i‐NPs)	Positive surface charge enables cartilage penetration; inhibits sPLA_2_ and NF‐κB	Preclinical (mouse OA models + ex vivo explants) [82]
Biomimetic scaffolds	Bilayered biomimetic scaffold (collagen/chitosan/silk fibroin)	Surface layer: KGN for chondrogenesis; transition layer: TGF‐β1 + aligned microtubules for migration/proliferation	Preclinical (in vitro + rabbit defect model) [83]
	Aragonite‐based biphasic scaffold (Agili‐C)	Supports cartilage repair; 78.7% defect fill rate at 2 years; hyaline‐like cartilage formation	Prospective multicenter clinical study (86 patients) [84]
Integrated strategies	Ginsenoside Rb1 hydrogel system	Inhibits PI3K/Akt, p38/MAPK, NF‐κB; reduces ROS and apoptosis; restores proteoglycan/COL2A1	Preclinical (rabbit KOA model) [85]
	Organ‐on‐a‐chip + SYN321 (uBeat MultiCompress)	Simulates OA mechanical loading; screens CECM intervention strategies in pathologically relevant conditions	Preclinical (in vitro) [86]

In summary, current therapeutic strategies for KOA have evolved into a comprehensive, multi‐dimensional system characterized by multi‐modal synergy. Traditional pharmacological approaches continue to advance through compositional optimization and mechanistic elucidation. Regenerative medicine, built upon stem cell and tissue engineering technologies, is developing rapidly. Gene editing and targeted therapies provide novel avenues for the precise regulation of CECM metabolism, whereas smart biomaterials offer innovative solutions for modulating the joint microenvironment and achieving structural regeneration. These strategies are not independent but demonstrate significant cross‐integration trends, exemplified by ‘stem cell‐biomaterial’ composite systems and ‘drug‐gene’ co‐delivery platforms. Their collective objective is to achieve precise regulation of the dynamic balance between CECM synthesis and degradation, thereby offering potential therapeutic prospects for delaying or even reversing the progression of KOA.

## 7 Future Perspectives

Despite remarkable advances in KOA therapeutic strategies targeting CECM homeostasis, considerable challenges persist, necessitating exploration across multiple research avenues with concrete scientific rationale.

Interdisciplinary integration and emerging technologies face several specific challenges that require systematic investigation. First, nanomaterial‐based targeted delivery systems, gene editing tools, and tissue engineering approaches, despite their immense potential, require comprehensive evaluation of long‐term biosafety profiles, immunogenicity, and in vivo metabolic clearance kinetics before clinical translation can be realized [82, 87]. Second, the development of smart biomaterials with pathological microenvironment responsiveness, such as the capacity to sense pH gradients, enzymatic activity, or redox states within osteoarthritic joints, represents a critical step toward achieving precise spatiotemporal control over drug and gene release [88], yet current iterations remain largely confined to proof‐of‐concept studies [82, 87]. Third, clinical translation efforts must prioritize both standardization and personalization of therapeutic protocols; most current stem cell therapies, biomaterials, and genetic interventions remain in preclinical or early‐phase clinical trials, underscoring the urgent need for harmonized cell manufacturing processes, material synthesis criteria, and treatment assessment frameworks [89]. The integration of multi‐omics technologies, including proteomics, metabolomics, and epigenomics, with artificial intelligence analytics offers a concrete pathway to identify stage‐specific molecular subtypes of KOA [90], thereby establishing a theoretical foundation for personalized precision medicine and enabling biomarker‐driven patient stratification in future trial designs [53, 55, 56].

Mechanistic investigation remains fundamental to uncovering novel therapeutic targets, and several specific research priorities can be delineated. Advanced biomimetic models, particularly organoids and organ‐on‐a‐chip platforms that recapitulate human joint biomechanical and biological microenvironments [91, 92], will be instrumental in deciphering the dynamic CECM‐chondrocyte interactome [86]. These platforms offer the unique advantage of incorporating physiologically relevant mechanical loading cycles, enabling researchers to distinguish between inflammation‐driven and mechanical‐driven degenerative pathways, a distinction that conventional monolayer cultures cannot resolve [86]. Emerging research domains focusing on programmed cell death modalities, such as pyroptosis and ferroptosis, senescence‐associated phenotypes, and epigenetic regulators such as SIRT6 activation, offer promising avenues for target discovery [93], with the mechanistic rationale being that these pathways operate upstream of overt matrix degradation and thus represent earlier intervention windows than currently available strategies [24, 25, 28, 29]. Furthermore, developing multi‐target combinatorial regimens is imperative. Given the limited efficacy of monotherapies against KOA's complex pathogenesis, future strategies should emphasize optimized combination therapies, such as stem cell‐gene engineering hybrids or bioactive factor‐loaded scaffold systems [94], that simultaneously modulate inflammatory milieus, enhance matrix anabolism, and suppress catabolic pathways to achieve synergistic therapeutic outcomes [76, 77, 83, 84, 85]. The scientific rationale for such combinations lies in the interconnected nature of KOA pathomechanisms, where targeting a single node is unlikely to overcome the network‐level compensatory responses that sustain disease progression [8, 10, 18].

In summary, through deepened mechanistic understanding, technological innovation addressing the specific challenges identified above, and accelerated clinical translation guided by standardized frameworks, CECM homeostasis‐targeted strategies hold potential to establish new paradigms for KOA disease modification and ultimately improve patient quality of life.

## 8 Abbreviations

A comprehensive list of abbreviations and their full terms is provided in Table S1.

## Author Contributions

**Chao Du:** conceptualization, methodology, writing – review and editing. **Jiwei Zhong:** data curation, investigation, formal analysis, writing – original draft. **Jialin Yang:** data curation, investigation, formal analysis, writing – original draft. **Yanbo Huang:** conceptualization. **Qi Liu:** data curation, investigation. **Dongdong Cao:** conceptualization. **Tianci Guo:** data curation, investigation. **Jida Wang:** conceptualization, writing – review and editing, methodology. **Aifeng Liu:** conceptualization, methodology, writing – review and editing.

## Funding

This work was supported by The National Natural Science Foundation of China (grant number 82474538), The Tianjin Health Commission Jinmen medical talent project (grant number TJSJMYXYC‐D2‐028), Research on the Mechanism of Medical Tai Chi in Regulating the Cartilage Repair Microenvironment for Knee Osteoarthritis by the Inheritance and Innovation Team of Traditional Chinese Medicine for Prevention and Treatment of Knee Osteoarthritis (2024GSZ03), Clinical Evaluation and Translational Research of Roujin Zhuanggu Granules in the Treatment of Knee Osteoarthritis (CXZJ2025004), Inheritance and Innovation Team of TCM for Prevention and Treatment of Knee Osteoarthritis (4042502041) and NATCM Initiative for Strengthening TCM Evidence‐Based Research, NATCM‐STER.

## Ethics Statement

The authors have nothing to report.

## Consent

The authors have nothing to report.

## Conflicts of Interest

The authors declare no conflicts of interest.

## Supporting information


**Table S1:** List of abbreviations.

## Data Availability

All data generated or analysed during this study are included in this published article and its Supporting Information files.
