# Supplementary material for: Cartilage Extracellular Matrix Homeostasis in Knee Osteoarthritis: Pathomechanisms and Therapeutic Targeting
Source: J Cell Mol Med. 2026 Oct 11;30(19):e71384. doi: 10.1111/jcmm.71384 (PMC13655213; doi:10.1111/jcmm.71384)
Supplement: Supplementary file 1 [file JCMM-30-e71384-s001.docx]

**Abbreviations**

**Supplementary Table S1.** List of Abbreviations

| **Abbreviation** | **Full Term** |
| --- | --- |
| ACLT | Anterior cruciate ligament transection |
| ADAMTS | A disintegrin and metalloproteinase with thrombospondin motifs |
| ADSCs | Adipose-derived mesenchymal stem cells |
| AKT | Protein kinase B |
| BM-MSCs | Bone marrow-derived mesenchymal stem cells |
| CECM | Chondrocyte extracellular matrix |
| COL2A1 | Type II collagen alpha-1 chain |
| COMP | Cartilage oligomeric matrix protein |
| CPCs | Chondrogenic progenitor cells |
| CS | Chondroitin sulfate |
| CT | Computed tomography |
| DAMP | Damage-associated molecular pattern |
| DKK1 | Dickkopf-1 |
| DMM | Destabilization of the medial meniscus |
| ER | Endoplasmic reticulum |
| FAK | Focal adhesion kinase |
| FAP | Fibroblast activation protein |
| GAG | Glycosaminoglycans |
| GDF5 | Growth differentiation factor 5 |
| GSH | Glutathione |
| HMGB1 | High mobility group box 1 |
| IGF-1 | Insulin-like growth factor-1 |
| iMSCs | Induced pluripotent stem cell-derived mesenchymal stem cells |
| KOA | Knee osteoarthritis |
| lncRNAs | Long non-coding RNAs |
| MAPKs | Mitogen-activated protein kinases |
| MCS | Microbially-derived chondroitin sulfate |
| MMPs | Matrix metalloproteinases |
| MRI | Magnetic resonance imaging |
| MSCs | Mesenchymal stem cells |
| PCD | Programmed cell death |
| PGE2 | Prostaglandin E2 |
| PI3K | Phosphoinositide 3-kinase |
| PRP | Platelet-rich plasma |
| PSP | Polygonatum sibiricum polysaccharide |
| qMRI | Quantitative magnetic resonance imaging |
| RAGE | Receptor for advanced glycation end products |
| RNAi | RNA interference |
| SASP | Senescence-associated secretory phenotype |
| SELM | Selenoprotein M |
| SIRT6 | Sirtuin 6 |
| SYSADOA | Symptomatic slow-acting drugs in osteoarthritis |
| TCM | Traditional Chinese Medicine |
| TGP | Total Glucosides of White Peony Capsule |
| TGF-β | Transforming growth factor-β |
| TLR4 | Toll-like receptor 4 |
| TMJOA | Temporomandibular joint osteoarthritis |
| TNF-α | Tumor necrosis factor-α |
| TRPV4 | Transient receptor potential vanilloid 4 |
| uCTX-II | Urinary C-terminal telopeptide of type II collagen |
| WJMSCs | Wharton's jelly-derived mesenchymal stem cells |
| ACLT | Anterior cruciate ligament transection |
| ADAMTS | A disintegrin and metalloproteinase with thrombospondin motifs |
| ADSCs | Adipose-derived mesenchymal stem cells |
| AKT | Protein kinase B |
| BM-MSCs | Bone marrow-derived mesenchymal stem cells |
| CECM | Chondrocyte extracellular matrix |
| COL2A1 | Type II collagen alpha-1 chain |
| COMP | Cartilage oligomeric matrix protein |
